# An integrated approach for mental health assessment using emotion analysis and scales

**DOI:** 10.1049/htl2.12040

**Published:** 2022-12-16

**Authors:** N. Shanthi, Albert Alexander Stonier, Anli Sherine, T. Devaraju, S. Abinash, R. Ajay, V. Arul Prasath, Vivekananda Ganji

**Affiliations:** ^1^ Department of Computer Science & Engineering Kongu Engineering College Perundurai Tamil Nadu India; ^2^ School of Electrical Engineering Vellore Institute of Technology Vellore Tamil Nadu India; ^3^ School of Computing and Creative Media University of Technology Sarawak Sarawak Malaysia; ^4^ Department of Electrical and Electronics Engineering Sree Vidyanikethan Engineering College Tirupati Andhra Pradesh India; ^5^ Department of Electrical and Computer Engineering Debre Tabor University Debre Tabor Ethiopia

**Keywords:** emotion recognition, face emotion recognition, mental health assessment, scales, speech emotion recognition

## Abstract

Depression is a prominent cause of mental illness, which could primarily increase early death. It is possible that this is the root of suicidal ideation, and it causes severe impairment in daily life. By detecting human face traits, artificial intelligence (AI) has cleared the road for predicting human emotions. This predictive technique will be used to conduct a preliminary assessment of depression. Prediction is accomplished using a mixture of four modules namely Facial Emotion Recognition (FER), Scales Questionnaire, Speech Emotion Recognition (SER), and Doctor Chat. FER2013 dataset is used for the FER module, while for speech‐based recognition, RAVDESS, TESS, SAVEE, and CREMA‐D are collectively used. To improve the accuracy of the FER, the people in the given image will be fed into a Face API created with TensorFlow JS, which will eventually be given to the proposed model that will recognize human faces in the image. For SER, a python library known as Librosa is used for extracting audio features and it will be fed to the proposed model. The scales module of the app has questionnaires that can be answered, and the result can be generated based on the scores obtained using established scales used in modern psychology such as the HAM‐D, YMRS etc., Though deep learning can predict emotions, the user may choose to speak with a real doctor about the issues to clear up any doubts. The application has a Doctor Chat module, which is essentially a chat bot for interacting with a doctor. Using this module, the users can talk, exchange files, and have their questions answered. The accuracy of FER is 91% whereas for SER, it is 82% on the test sets. The proposed approach produces the highest accuracy for the benchmark dataset. These four modules will work together to produce a homogenous depression report.

## Graphical abstract

For leading a healthy life, a person's mental state is critical. Depression is a prominent cause of mental illness, which could primarily increase early death. It's possible that it's the root of suicidal ideation, and it causes severe impairment in daily life as a result. By detecting human face traits, artificial intelligence has cleared the road for predicting human emotions. This predictive technique will be used to conduct a preliminary assessment of depression or anxiety. Prediction is accomplished using a mixture of four modules: Facial Emotion Detection, Scales Questionnaire, Speech Emotion Detection, and Doctor Chat. For the facial emotion identification module, the FER2013 dataset is employed, while for speech‐based detection, RAVDESS, TESS, SAVEE, and CREMA‐D are combined. To improve the accuracy of the forecast, the people in the given image will be fed into a Face API created with TensorFlow as the backend, which will eventually recognize human faces in the image. A library known as Librosa supports speech‐based detection. Because it is mobile and can be accessed by anybody via a browser, the entire process is interfaced using a Web Application created with Vue JS and backed by Node. The Scales module of the app has questionnaires that can be answered, and the result can be generated based on the scores obtained using established scales used in modern psychology such as the HAM‐D, YMRS, etc., Though deep learning can predict emotions, the user may choose to speak with a real doctor about the issues in order to clear up any doubts. The application has a Doctor Chat module, which is essentially a chat bot for interacting with a doctor. Users can talk, exchange files, and have their questions answered. The accuracy of FER is 91% whereas for SER, it is 82% on the test sets. These four modules have been integrated and can operate together to detect and analyze depression early on, thereby reducing effects of the same.

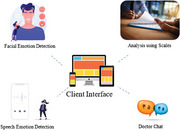

## 1 INTRODUCTION

A person's mental health is critical to live a happy life. People in today's environment are preoccupied and rarely devote time to self‐care. This puts them at a higher risk of becoming stressed. Depression is the leading cause of poor mental health and raises the risk of suicidal ideation. Preliminary depression study and diagnosis will save many lives, as about 8 million individuals die by suicide each year [1]. Psychology is not technically equipped to deal with many patients. As a result, it is critical to locate a solution that is dependable, mobile, and secure. A psychiatrist needs a preliminary assessment report for a person's mental health to prepare themselves to treat patients by gaining insights. This approach will considerably minimize time and resource‐intensive manual labour, and will benefit both the doctor and the patient as it is easily accessible and reliable.

The existing system suffers with issues like reliability, standardization, reluctance, and less accuracy. Since hospitals tend to use bespoke software, the existing system has greater difficulties than standardized programs. Many psychiatrists still conduct question‐and‐answer sessions with patients, which is time intensive for the patients. The hospitals use different methodologies for detecting depression, the data from different hospitals will differ, which will have a substantial impact on the report's credibility [2].

To achieve consistent results, the entire procedure must be standardized and integrated into the hospital's management system. People who are depressed are hesitant to see a doctor and seek counseling. They usually want confirmation on their health, which is where this app comes in handy. Even though the legislation protects the user's data that he or she shares with the doctors, the information is eventually put into their internet‐based software [3], which is particularly vulnerable to data breaches. The solutions that have already been created to meet the demand are less accurate and have reliability difficulties. The modules were not combined to produce better outcomes, and the model's accuracy was inadequate in comparison to the system's capabilities [4]. Due to these issues, there is no standardized system at present for depression analysis. Thus in the proposed methodology, Facial Emotion Recognition (FER), Speech Emotion Recognition (SER), and scales are combined to predict the state of mental health in order to produce a more accurate result.

## 2 RELATED WORKS

Many researchers are working on mental health assessment in recent years. In this section, some of the recent studies on mental health assessment are presented. Mellouk et al. [5] proposed emotion recognition on face expressions as an attractive study field. Mollahosseini [6] offers a deep CNN for FER datasets. They utilized three CNN with the same architecture. Each one detects a region of the face, such as the mouth, eyebrow, eye, and ears. In 2019, Agrawal and Mittal [7] studied the influence change of the CNN parameters on the recognition rate using the FER2013 database. According to the investigations, researchers create models of CNN and acquire an average of 65.77% of accuracy.

Tarnowski and Paweł [8] showed the findings of recognising seven human facial expressions. A person's mental health relates to his/her cognitive, behavioural, and emotional well‐being. Applications utilising machine learning (ML) and artificial intelligence (AI) algorithms may foresee such mental states and operate as a monitor for any aberrant behaviour in individuals. Longo et al. [9] present the creation and validation of a new well‐being questionnaire. Digital signal processing is a matter of great interest, and experts have come up with several techniques for recognizing speech emotions. The SER task is subdivided into two phases: the selection of dominant acoustic features and the categorization of emotions [10]. In SER, robust feature selection is a difficult problem. Researchers have derived various features in the area of speech processing, including prosodic features, source‐based excitation features, vocal traction factors, and other hybrid features [11].

Based upon the number of acoustic parameters employed and statistical variability of these parameters, the number of features extracted by acoustic analysis may reach very high levels. Not all of these features are useful for emotion recognition, and various emotions may affect different vocal characteristics. As a result, feature selection techniques are utilized to improve emotional recognition performance while reducing effort by reducing the number of features [12].

Researchers have used AI and DL techniques to improve the Human Computer Interface, which includes emotion identification in voice signals, as technology has progressed. In this sector of study, researchers have created novel techniques for efficient SER using AI and DL [13, 14, 15, 16, 17]. However, there are promising possibilities and rich ground for future research opportunities [18, 19, 20]. Neural networks' layer‐wise structure adaptively learns characteristics from available raw data in a hierarchical manner [21]. CNNs are often employed to improve data categorization and pattern identification. The input data is processed in the form of receptive fields by these networks, which feature small size neurons on every layer of the chosen model architecture [22].

Zhao et al. recognized speech emotion using 1‐D and 2‐D CNNs with a Long Short‐Term Memory (LSTM) network [23]. While the majority of SER systems use CNNs, at least one LSTM network is added to cope with temporal dependencies and spectrum changes; nonetheless, this adds depth and complexity to the system. Rather than that, Kim et al. presented a three‐dimensional CNN for learning spectro‐temporal characteristics [24]. Table 1 gives the existing studies on emotion recognition in literature.

**TABLE 1:** Existing studies on emotion recognition

**Author**	**Architecture used**	**Database used**	**Accuracy**
Manav Pradeep Jain et al. [3]	CNN	FER2013	63.2%
Tarnowski and Pawel [8]	k‐NN classifier and MLP neural network	KDEF database	95.5% and 75.9% respectively
Özseven [12]	SVM, k‐NN, MLP, PCA	EMO‐DB, eNTERFACE05, EMOVO, and SAVEE	62%, 69.23%, 60.40%, 72.39%, respectively
Albornoz et al. [16]	Restricted Boltzmann machines and deep belief networks	German emotional database	69.14%
Akhand et al. [25]	DCNN	KDEF and JAFFE	96.51% and 99.52%, respectively
Deepak et al. [26]	DCNN	CK+ and JAFFE dataset	95.23% and 93.24%, respectively
Xi Zhou et al. [27]	Stacked autoencoder network, deep belief network	German Berlin Emotional Speech Database	65%
Kun Han et al. [28]	Extreme Learning Machine	Interactive Emotional Dyadic Motion Capture (IEMOCAP)	54.3%

From the literature review, the following conclusions are made,

- The developed model in the literature has lesser accuracy
- No Integrated System
- No Multi‐model approach

Thus, building an application to cater to this need is essential for improving the overall experience of patient and doctors in psychology. The integration of FER, SER, Scales, and doctor chat is done for the first time to enhance the accuracy of mental health prediction.

## 3 MATERIALS

This section presents the various datasets used in the proposed approach.

### 3.1 FER2013

FER2013 is an open‐source dataset uploaded publicly for a Kaggle Competition. This dataset consists of 35,887 greyscale, 48 × 48 pixeled face photos with seven different emotions. It was set to accessible to everyone after completing the competition. This dataset is considerably preferred over the other datasets [29] that does the same thing like, AffectNet, Ascertain, Dreamer, CK+, Emotic, and K‐EmoCon. The benefit with FER2013 is it has a smaller number of Japanese and Chinese faces. It is a significant point to consider as those people have slightly different facial features compared to the rest.

### 3.2 Depression rating scales

Psychiatrists use depression rating scales as one best way to assess the nature and severity of patients’ mental health and monitor their clinical progress [9]. Depression rating scales are a systematic method for determining the intensity of depression symptoms over time. Researchers can judge and grade a patient using these depression rating scales by considering the observed characteristics.

### 3.3 CREMA

CREMA‐D is a collection of 7442 unique clips featuring 91 actors. These recordings include 48 male and 43 female actresses ranging in age from 20 to 74 and representing a range of races and ethnicities.

### 3.4 RAVDESS

RAVDESS has 1440 records, 60 sessions per actor multiplied by 24 actors equals 1440. It is made of 24 professional actors (12 female and 12 male), each of whom speaks two lexically related phrases in a neutral North American accent.

### 3.5 TESS

TESS includes an audio of two actresses (age 26 and 64 years) expressing each of seven moods using a collection of 200 target words in the carrier phrase ‘Say the word’. There is a sum of 2800 data points (audio files).

### 3.6 SAVEE

SAVEE has been built as a prerequisite for constructing an autonomous emotion identification system. The collection features recordings of four male performers portraying seven unique moods, totaling 480 British English words.

## 4 PROPOSED METHODOLOGY

**FIGURE 1:**
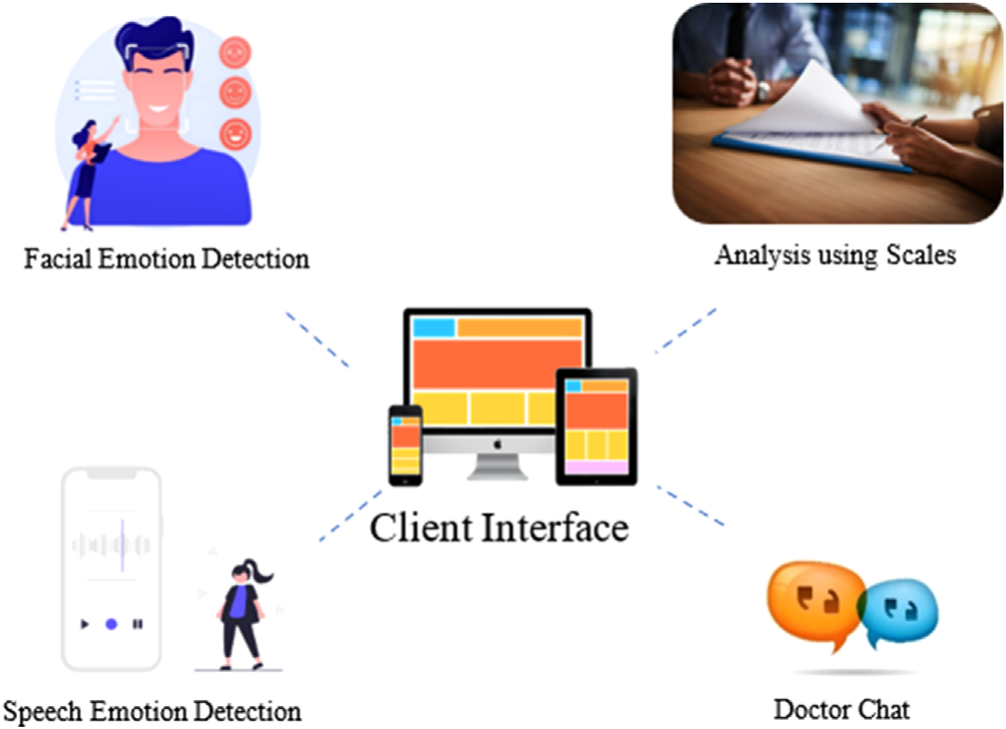
System architecture

The major components of the proposed paper comprise four modules namely FER, Scales Questionnaire, SER, and Doctor Chat. The visual representation of the system architecture is shown in Figure 1.

### 4.1 Facial emotion recognition

**FIGURE 2:**
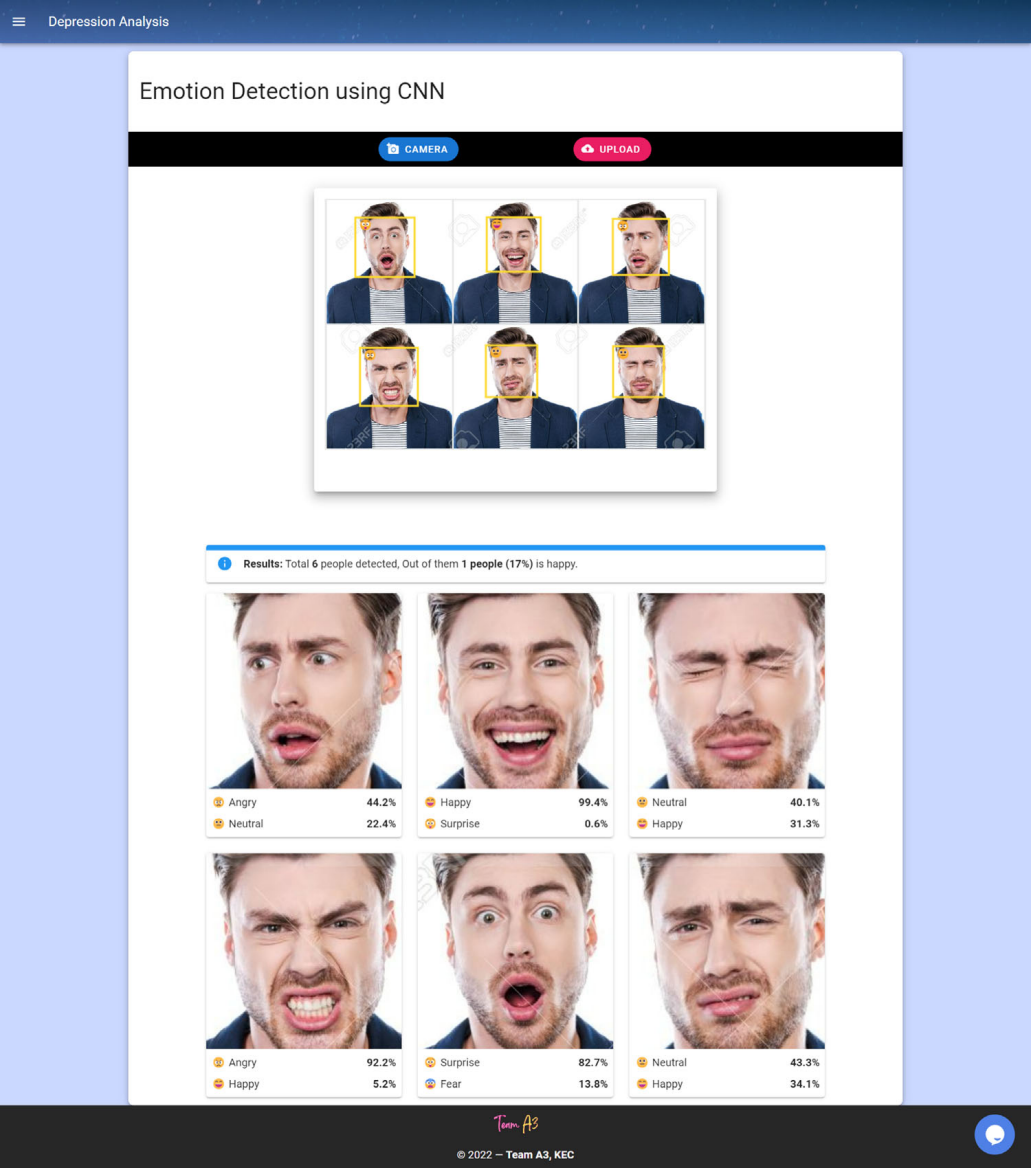
Facial emotion recognition module

The client can use this module to analyze their facial emotion. This module can function as a preliminary depression screening test. The user can either upload their photo from their local storage or capture a live photo using their web/mobile camera. This uploaded image is processed with the pre‐trained model embedded in the web application. The analysis result is displayed, and the canvas is drawn over the uploaded image. The facial emotion module interface is shown in Figure 2.

Once the client uploads the image, the uploaded image is converted to greyscale. It is because grey‐scale pictures are enough for many tasks, negating the need for more intricate and difficult‐to‐process colour photos. This grey‐scaled image is pixelated and resized into a 48 × 48 image pixel array. The face‐api.js [30] JavaScript package incorporates CNNs, offers a solution for face identification recognition, and faces landmarks. Also, face‐api.js employs TensorFlow.js [31] and is designed for the mobile, desktop, and web. Using face‐api.js, faces on this resized array are detected and cropped out. This resized pixel array is fed as input for the pre‐trained model.

**FIGURE 3:**
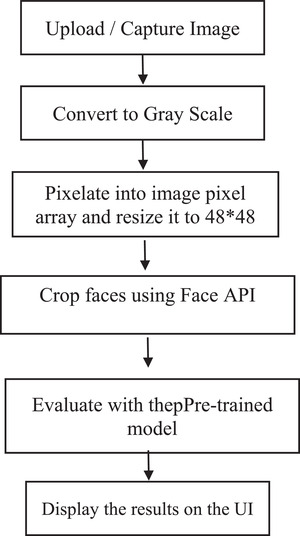
Facial emotion recognition flow

The pre‐trained model processes this array to predict the outcome based on its previous learnings. The results are displayed with the top two emotions with their percentage of match detected from the face, and canvases are drawn over the faces depicting their facial emotion. This FER workflow is shown in Figure 3.

#### 4.1.1 Data pre‐processing

The data obtained from the FER 2013 dataset is pre‐processed. The dataset contains two different attributes, and one is a pixel value of a 48 × 48 grey‐scaled image and the other attribute is its corresponding emotion. It is a label encoded value in the range of 0 to 6 representing seven different emotions namely 0 → Angry, 1 → Disgust, 2 → Fear, 3 → Happy, 4 → Sad, 5 → Surprise, 6 → Neutral. These are the features with which the model is trained. 80% of the dataset is used for training and 20% is used for testing.

**FIGURE 4:**
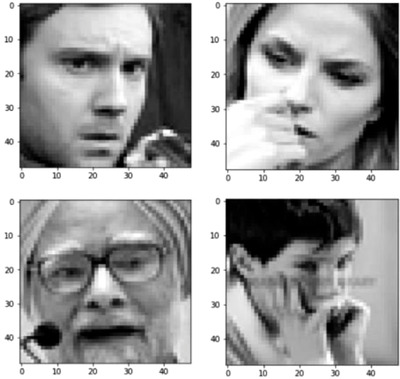
Grey‐scaled image in the dataset

Among the 35,887 records in the dataset, 28,709 records are used for the training purpose and 3589 records each for both private and public tests. The images are visualized from dataset pixel using pyplot [32] python library. The plotted, grey‐scaled images are shown in Figure 4. The dataset is ready now from a theoretical perspective; however, to utilize this dataset with the python functions, the data has to be normalized. The purpose of normalization is to transform the values of numeric columns in a dataset to a standard scale without losing information or introducing disparities in the ranges of values. Thus, the image pixel array is normalized with its pixel value in the range of 0 to 1. The feature and the label are split and made ready for further processing with the model.

#### 4.1.2 Building and training the model

The model is developed and trained in the python programming environment using various libraries like Pandas, Keras, Numpy, and Matplot. The Keras module provides the functions for implementing the sequential neural network. Keras enables us to construct a layer‐by‐layer model. The Keras ImageDataGenerator is used to rotate, zoom, and manipulate the original picture. Because the learnt model is trained on numerous variants of the same picture, the number of photos in each epoch is equal to the number of original images. As a result, the learnt model may be more resilient and accurate. When a metric stops improving, reduce learning rate is used to slow down the learning rate. Models often benefit from reducing the learning rate by a factor of 2 to 10 once learning stagnates. This callback tracks a quantity and reduces the learning rate if no progress is observed after a predefined number of epochs.

In the sequential model, a two‐dimensional convolutional layer is added, and this layer creates a convolutional kernel, which winds with layer's input which helps to produce a tensor of outputs. The MaxPooling 2D layer down‐samples the input representation by taking the maximum value throughout the window defined by pool size for each dimension along the feature axis. The window is shifted by strides in each dimension and added two times 2D convolutional layer with activation as RELU and again added MaxPooling layer with pool size as (2, 2). By adding a flattened layer, it turns the data into a one‐dimensional array for entering it to the subsequent dense layer. Then the output of the convolutional layers is flattened to generate a single long feature vector; then, it is merged with four dense layers to the model. Finally, the model is compiled with binary cross‐entropy for loss calculation and Adam as optimizer [25]. After constructing the model, it is trained using fit generator, wherein the ImageDataGenerator will apply filters like zooming, rotating, flipping etc., over the image in each epoch.

### 4.2 Depression rating scales

**FIGURE 5:**
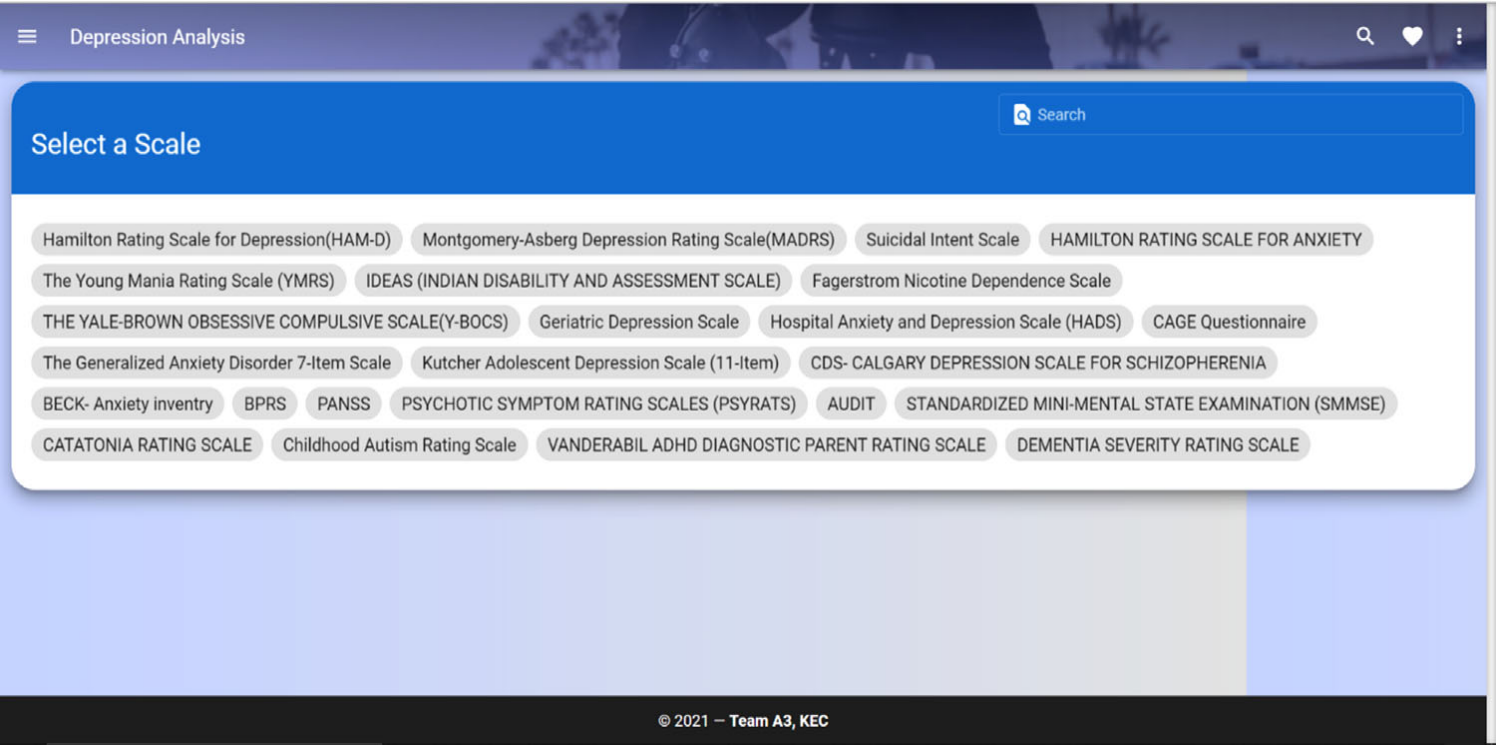
Analysis using scales module

The user can use scales module to analyze the severity of depression. This can act as a preliminary depression screening test. At the same time, this module can be used to make a judgment on whether the patient needs to be assessed for further treatment and medication or not. Once the user opts for this module, a list of standardized scales will be displayed. The user must choose the appropriate scale based on the undergoing problem. On selecting a specified scale, the descriptive scale phrases with their appropriate options are displayed one after the other. These scales are chosen based on the discussion with doctors who are practicing mental health detection for a long time. The analysis using scales module interface is shown in Figure 5.

Each option will have varied scores. Options representing higher severity will have higher scores, and similarly, lower‐severe options will have lower or least scores. After answering all the questions on a specified scale, the total scores are tallied and displayed as an outcome of this module. Each scale will have its range indicating the severity level. Based on the score obtained from answering a specified scale, severity of depression levels like Moderate or Severe is shown as a result.

### 4.3 Speech emotion recognition

Speech is one of the most natural ways for humans to express themselves. It provides information about the speaker, the language, and his or her emotions. Due to the critical role emotions play in communication, detecting and analyzing them is crucial in today's digital age of distant communication. Thus, SER means understanding the emotional state of a human by extracting or detecting features from his/her voice. Due to the subjective nature of emotions, it is difficult to identify them. There is no universally accepted method for quantifying or classifying them.

#### 4.3.1 Feature extraction

Speech is a complicated three‐dimensional signal in which three axes represent time, amplitude, and frequency. Feature extraction in speech refers to extraction of desirable features from the human voice to detect the emotion of a human. There are three categories of characteristics in a speech: lexical features (vocabulary used), visual aspects (the speaker's expressions), and acoustical features (sound properties like tone, pitch, jitter etc.). Acoustic features are mainly categorized into prosodic features and **Cepstral** features [33, 34].

#### 4.3.2 Mel frequency cepstral coefficients (MFCCs)

One of the most effective tools for extracting features from audio waveforms is MFCC. For each audio signal sample, the MFCC approach produces 39 features that are fed into the speech recognition model. The feature count is sufficient to learn the audio's information. The amplitude of frequencies is determined by 12 parameters. It gives an adequate number of frequency channels for audio analysis.

#### 4.3.3 Data pre‐processing and data augmentation

The CREMA, SAVEE, TESS, RAVDESS datasets are combined for SER. Calm emotion records are merged with neutral records because calm emotion records are very minimal when compared to other emotions and often get confused with neutral emotion based on learnings from confusion matrix. This combined and processed dataset is used as the source for training the model. Data augmentation is the process of creating new simulated training samples from our original training set by introducing minor changes. The goal is to make our model invariant to such perturbations and to improve its generalizability. Data augmentation techniques like injecting noise, stretching, pitching, shifting, higher and lower speed are used. Using python library Librosa, MFCC features are extracted from the audio files. These features are then normalized using standard scalar.

#### 4.3.4 Multi‐model approach

Multiple model approach is adopted here to improve the model prediction over most confused outcomes. This approach is adopted to SER. From the confusion matrix, it can be observed that the model has a number of false‐positive and false‐negative values significantly higher in these categories, Angry–Happy (158), Sad–Disgust (136), Fear–Sad (179). Neutral is being confused with almost all the emotions which are acceptable based on the nature of the same. Thus, to improve the accuracy of the model, three different models (the same SER model which has been trained with only the specific emotion's dataset) have been created for the above‐mentioned three confusion groups. For instance, the Angry–Happy model is trained with the dataset consisting of those two emotions. The accuracies of these three models are,

- Fear–Sad Model: 93.17%
- Sad–Disgust Model: 92.01%
- Angry–Happy Model: 91.54%

**FIGURE 6:**
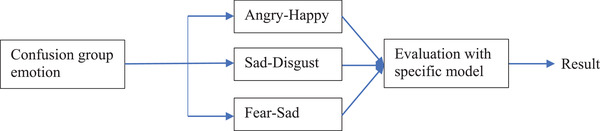
Multi model approach

If the SER model outputs the top two emotions falling in the above categories, the audio will be further processed with the respective model for classification. Upon validation, it is observed that the multi‐model approach can improve the overall working of the SER process thereby achieving 90% accuracy. Figure 6 clearly shows the multi‐model approach proposed in the paper.

### 4.4 Doctor chat

**FIGURE 7:**
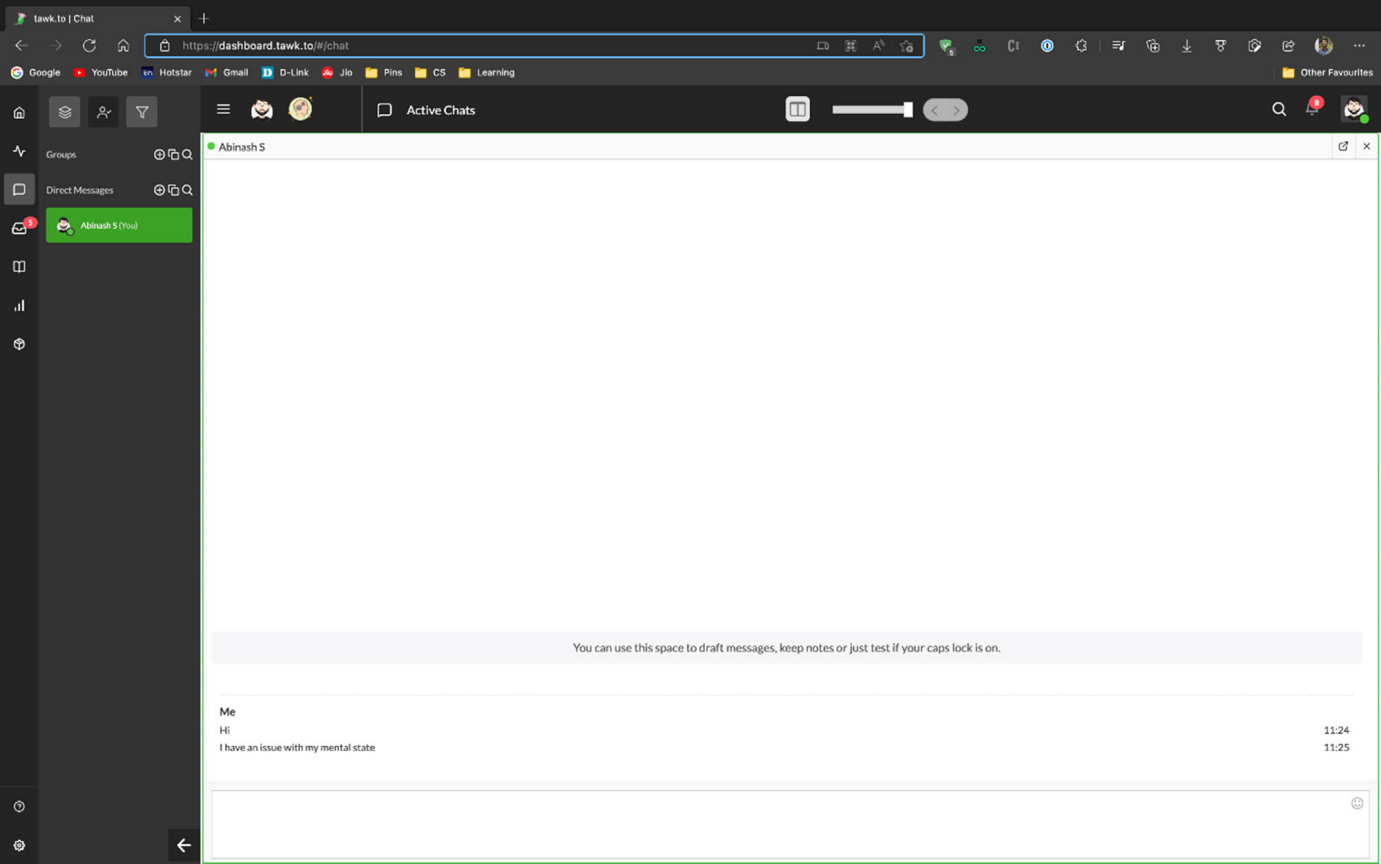
Doctor dashboard

The doctor chat module is available for people who want to consult a psychiatrist for further analysis of depression. This would be helpful with the other models as they will act as a preliminary analysis tool and the doctor can clear up further confusions. This chat process is supported by Tawk.to platform which is a live chat plugin which can be added to any website. It also features an interactive dashboard with features like chat responding, ticket creation, Internal chat with fellow doctors, chat export, and mailing for the use of doctors. Users can chat with the doctor by entering basic details like name, age, and gender. They can also share photos and save the chat transcript through emails. The benefit of using external platform like Tawk.to is GDPR and FERPA compliant. Being a critical application handling very sensitive user information, data security is the high priority. All the models FER and Scales, the data never leaves the user system. The model execution and result calculations are done in the users local machine. For SER, the voice data is processed in AWS Lambda. It will not carry any other user information meta data as well as to ensure security, the data is encrypted both in transit (using HTTPS) and at rest (using AWS Key Management Service). This will ensure the privacy of the users and their data [35]. The doctor chat interface is displayed in Figure 7.

### 4.5 Integration

To achieve a more distinctive prediction, it is necessary to integrate all four modules together. Many existing systems for detecting depression are lagging at integration which was making the system unusable. Here, the integration process has different stages of score calculation.

The emotion results of the FER and SER modules are converted into scores with respect to the emotion given in Table 2. Each scale has different weightage of their own. But for making the calculation effective, the scale's range has been normalized to the value of 100. So, if the user chooses any scale of their choice, the scale range will not affect the final calculation of depression level.

**TABLE 2:** Score calculation

**Emotion**	**Score**
Sad	100
Angry	90
Disgust	80
Fear	70
Neutral	50
Happy	25
Surprise	10

**FIGURE 8:**
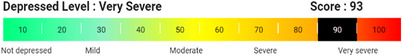
Integrated result

**FIGURE 9:**
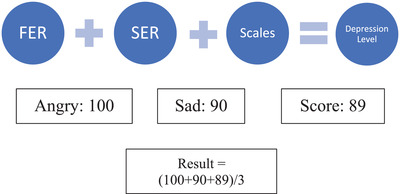
Score calculation process

The score of scales is also converted into 100 bases. The final score is calculated by taking an average of the three scores which will be compared with the result range as shown in Figure 8. For example, if the result of FER is Angry, and the result of SER is Sad, the corresponding score would be 100 + 90 = 190 and if the score of scales module is 89, the final score of the depression would be 93. Comparing 93 to the result range, it will be predicted as Very Severe depression. The process is displayed in Figure 9.

### 4.6 Deployment

The entire application is deployed in AWS, frontend is powered by Amplify. For FER, all the backend functions are deployed in the Lambda using code. For SER, a docker image is created with all the required libraries and packages that are needed to run the python model and push it to the AWS Elastic Container Registry. SER function is created in the lambda by using the SER docker image. The SER model requires .wav file to process the result, but the audio recorded in the browsers is in the ogg audio format. To convert the ogg into a wav format, a separate lambda function is created to perform the conversion. So, when the input file is in the ogg format then the nested lambda function will be followed to convert the file into wav audio format and then given it as an input to the SER function. API Gateway is a suite of tools for building and documenting web APIs that send HTTP requests to Lambda functions. So, with the help of API Gateway the frontend can send a request and receive a response from the lambda function.

## 5 RESULTS AND DISCUSSION

The FER model was able to give only 65% accuracy on the test set. To reduce overfitting, LR on Plateau, image preprocessing, and dropouts are used. LR Reduce on Plateau is a regularization process. After completing each epoch, the call back will be called, in which, if there is no improvement in the accuracy, the learning rate will be reduced accordingly. The image processing was taken care of by the Keras inbuilt Image Data Generator which has different attributes which need to be tuned accordingly for the dataset. After applying these techniques, the model was able to produce 91.3% accuracy with just 13 epochs. Using multiple convolution layers was also one of the reasons for this higher accuracy.

It is the best test accuracy for the FER2013 dataset. Though the accuracy is better, to improve the in‐app performance of the model, Multi‐Task Cascaded Convolutional Neural Networks (MTCNN) is used for face recognition. By achieving ensemble learning methodology, the output of the MTCNN model is being fed to the exported model. The results will be shown within seconds compared to primitive methods that would take minutes to recognize the emotion.

Analyzing the model's performance, the model accuracy was 85% at the first epoch and constantly increases until it saturates at the 13th epoch. After which, the learning rate is reduced to avoid overfitting. At the end of the 13th epoch, the training accuracy is approximately 91%, and the test accuracy is 90.3%. The mean squared loss, at last, was 0.2190, which is significantly less compared to other models built for the same dataset.

For scales, it gave accurate results by comparing the cumulative score from the user with predefined ranges. As the data were fetched from standard websites, it reproduced the same set of results for all possible total values.

**FIGURE 10:**
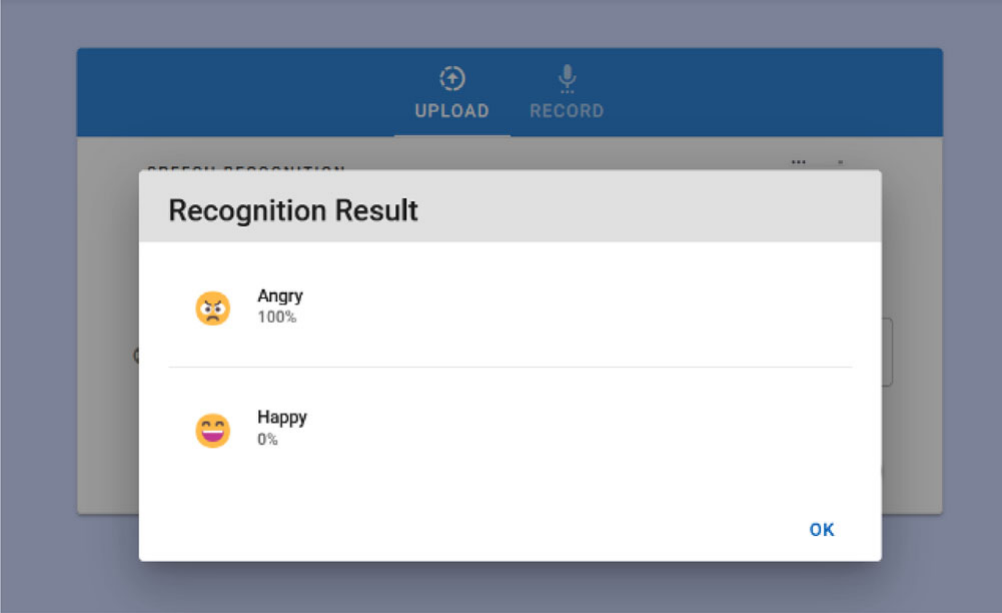
SER result interface

**FIGURE 11:**
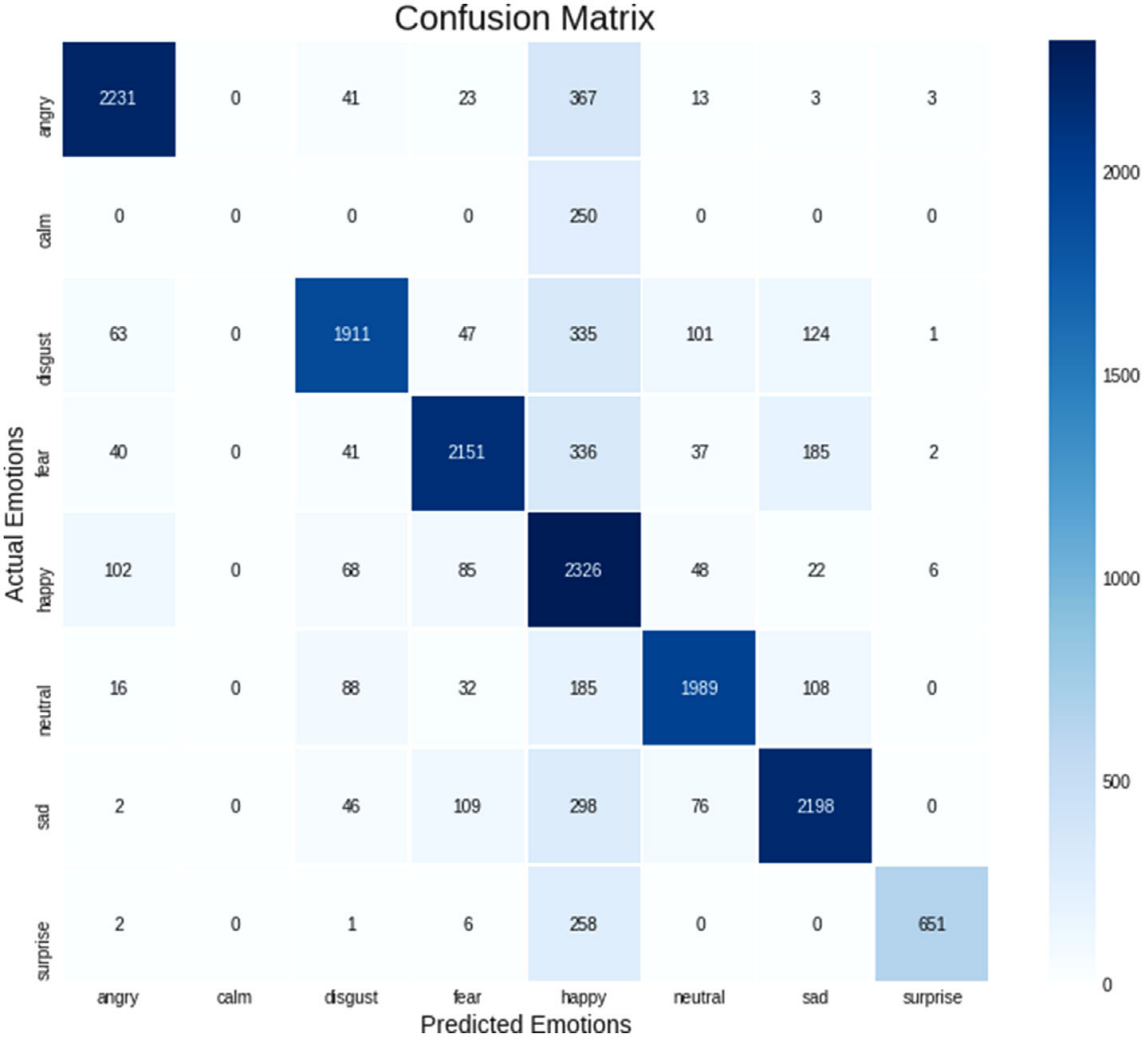
SER confusion matrix

The SER result is shown with the top two emotions of the uploaded or recorded audio file as indicated in Figure 10. The SER model performed well with a train accuracy of 87.94% and validation accuracy of 82.42%. The confusion matrix of the SER model is shown in Figure 11.

**FIGURE 12:**
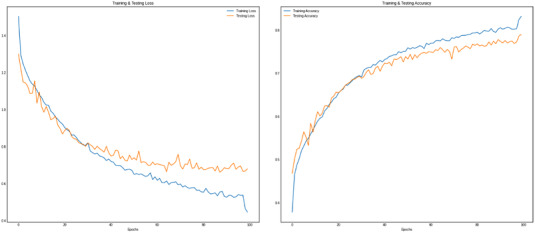
SER accuracy graph

As stated earlier, the ensemble learning methodology is adopted as there was more confusion in the three identified groups of emotions. Neutral emotion is confused with every other emotion, and it is acceptable due to the nature of the same. The growth of accuracy and withering of loss is shown in Figure 12.

Table 3 displays the precision, recall, and the F1 score of the SER model. F1 score of all the emotions is above 0.8 but neutral which is expected to be the reducing factor of the overall validation accuracy of the model. Same is the case for both precision and recall producing overall model accuracy of 82%. With multi‐model approach, it can be increased to 90%.

**TABLE 3:** F1 score analysis of speech emotion recognition model

Emotions	Precision	Recall	F1‐Score	Support
ANGRY	0.93	0.84	0.88	2681
DISGUST	0.88	0.79	0.83	2582
FEAR	0.91	0.80	0.85	2792
HAPPY	0.89	0.81	0.85	2657
NEUTRAL	0.58	0.92	0.71	2668
SAD	0.90	0.82	0.86	2729
SURPRISE	0.99	0.71	0.83	918
ACCURACY			0.82	17,027

The results are consolidated in Table 4 which has different methods of CNN model building from different approaches that shows the highest accuracy has been achieved through the proposed approach given in this paper.

**TABLE 4:** Procedural comparison

1D CNN of simple MFCC mean	45%
1D CNN of simple MFCC mean with data augmentation	48%
2D CNN of entire MFCC without augmentation	64%
2D CNN of entire MFCC with augmentation	60%
2D CNN of entire Log‐melspectogram without augmentation	63%
2D CNN of entire Log‐melspectogram with augmentation	63%
**1D CNN of entire MFCC with data augmentation**	**82%**

The doctor chat is able to work well with both the mobile and desktop interfaces which will eventually help the users for seamless communication for professional help over online. The integration is able to give a clear‐cut depression level results which is understandable by naïve users. A team of psychologists and psychiatrists are consulted for validating the final Depression Level calculation from all the four modules. It is being done by their recorded experience in seeing depression patient's top emotional showcase. The emotions are ordered in the suggested manner and an arbitrary value of 0 to 100 has been set for mathematical calculation of depression levels.

## 6 CONCLUSION

This work provides a person with the interface to identify the person's current mental state by capturing the face via the camera or uploading the image from the local storage. Once the image is uploaded, it is converted to greyscale. Using face‐api, faces on the grey‐scaled image are detected and cropped out. The pre‐trained model processes this array to compute the result based on its learning.

Depression rating scales are the standardized way to measure and evaluate the severity of depression during a period. After answering all the questions on a specified scale, the total scores are calculated and displayed as an outcome of this module. Each scale will have its range indicating the severity level. Based on the score obtained from answering a specified scale, severity of depression levels like moderate or severe is shown.

SER and Doctor chat would improve the working of the system by adding two more prediction methodologies. SER operates on voice prediction using multiple datasets and uses ensemble learning to improve accuracy. Doctor chat is useful for instant communications with professionals through the app itself.

The aim of this application is to be a preliminary assessment tool for depression handling which should be applicable for both the doctors and patients. There is not anything which integrates different approaches of depression prediction and provides an integrated result as this application does.

## AUTHOR CONTRIBUTIONS

Shanthi N & Albert Alexander Stonier: Conceptualization, Methodology, Software. Anli Sherine, T. Devaraju & Abinash S : Data curation, Writing‐ Original draft. Ajay R, Arul Prasath V & Vivekananda Ganji : Visualization, Investigation.

## CONFLICT OF INTERESTS

The author(s) declare(s) that there is no conflict of interest.

## Data Availability

The data that support the findings of this study are available from the corresponding author upon reasonable request.
